# Smooth muscle-specific HuR knockout induces defective autophagy and atherosclerosis

**DOI:** 10.1038/s41419-021-03671-2

**Published:** 2021-04-09

**Authors:** Shanshan Liu, Xiuxin Jiang, Xiuru Cui, Jingjing Wang, Shangming Liu, Hongxuan Li, Jianmin Yang, Cheng Zhang, Wencheng Zhang

**Affiliations:** 1 The Key Laboratory of Cardiovascular Remodeling and Function Research, Chinese Ministry of Education, Chinese National Health Commission and Chinese Academy of Medical Sciences, The State and Shandong Province Joint Key Laboratory of Translational Cardiovascular Medicine, Department of Cardiology, Qilu Hospital, Cheeloo College of Medicine, Shandong University, Jinan, China; 2 Cardiovascular Disease Research Center of Shandong First Medical University, Central Hospital Affiliated to Shandong First Medical University, Jinan, China; 3 Department of General Surgery, Qilu Hospital of Shandong University, Jinan, China; 4 Department of Physiology and Pathophysiology, School of Basic Medical Sciences, Shandong University, Jinan, China; 5 Department of Histology and Embryology, School of Basic Medical Sciences, Shandong University, Jinan, China

**Keywords:** Cardiovascular diseases, Pathogenesis

## Abstract

Human antigen R (HuR) is a widespread RNA-binding protein involved in homeostatic regulation and pathological processes in many diseases. Atherosclerosis is the leading cause of cardiovascular disease and acute cardiovascular events. However, the role of HuR in atherosclerosis remains unknown. In this study, mice with smooth muscle-specific HuR knockout (HuR^SMKO^) were generated to investigate the role of HuR in atherosclerosis. HuR expression was reduced in atherosclerotic plaques. As compared with controls, HuR^SMKO^ mice showed increased plaque burden in the atherosclerotic model. Mechanically, HuR could bind to the mRNAs of adenosine 5′-monophosphate-activated protein kinase (AMPK) α1 and AMPKα2, thus increasing their stability and translation. HuR deficiency reduced p-AMPK and LC3II levels and increased p62 level, thereby resulting in defective autophagy. Finally, pharmacological AMPK activation induced autophagy and suppressed atherosclerosis in HuR^SMKO^ mice. Our findings suggest that smooth muscle HuR has a protective effect against atherosclerosis by increasing AMPK-mediated autophagy.

## Introduction

Atherosclerosis is a chronic and systemic vascular inflammatory process, the pathological basis of coronary artery disease, myocardial infarction, and stroke^1^. Despite many treatment options, it remains the leading cause of death worldwide. Developing new strategies to prevent plaque formation and rupture has become an important research area. Atherosclerosis is initiated by endothelial dysfunction and vascular inflammation caused by cardiovascular risk factors such as hyperlipidemia and hypertension. Lipoproteins in the blood enter the arterial wall from the damaged endothelial cells^2,3^. Inflammatory factors can stimulate monocytes and vascular smooth muscle cells (VSMCs) to engulf oxidized low-density lipoprotein (ox-LDL) and form foam cells^4^. Disordered lipid metabolism, inflammation, and endothelial injury all seem to play major roles in atherosclerosis^4,5^. However, the pathogenesis of atherosclerosis is still unclear and needs further study.

VSMCs are crucial in atherosclerosis. Most foam cells in early atherosclerosis are derived from VSMCs^6^. Aberrant proliferation and migration followed by phenotypic switching of VSMCs are involved in the formation of atherosclerosis^7^. VSMCs produce the extracellular matrix, which forms the fibrous cap to prevent plaque rupture^8^. The death and senescence of VSMCs participate in the formation of atherosclerotic plaque and also promote the instability of plaque in advanced lesions^7,9^. However, the specific regulatory mechanism of VSMCs in atherosclerosis is not clear.

Autophagy is an essential subcellular process that has a “housekeeping” role in the normal physiological functions of the body. Autophagy participates in numerous physiological and pathological processes, including cell differentiation, growth regulation, aging, immunity, and tumor suppression^10^. Autophagy is a multi-step process that requires a variety of autophagy-related proteins that take part in nucleation, expansion, and finally fusion with lysosomes of autophagosomes^11,12^. Accumulating evidence suggests that autophagy is involved in the occurrence and development of atherosclerosis and related diseases^13,14^. Smooth muscle-specific Atg7 knockout can promote atherosclerosis^15^, but the molecular mechanism of autophagy in atherosclerosis still needs elucidation.

Human antigen R (HuR) is a ubiquitous and conserved RNA-binding protein that binds to AU-rich elements (AREs) and alters ARE-mediated mRNA turnover and translation^16^. HuR binds to an extensive list of RNAs that participate in cell proliferation, apoptosis, and differentiation^17,18^. Therefore, HuR may have an important effect on both pathologic and physiologic functions. Although cancer is the most widely studied disease associated with HuR^19^, the molecule is also reported to take part in chronic inflammation and nervous system diseases^18,20^. Our recent work showed that adipose tissue-specific HuR knockout caused obesity and metabolic disorders^21^. Also, knockout of HuR enhanced VSMC contraction and hypertension^22^. However, the role of HuR in autophagy and atherosclerosis remains unclear.

In this study, we examined HuR expression in atherosclerotic plaques and found its expression decreased. Using smooth muscle-specific HuR knockout (HuR^SMKO^) mice, smooth-muscle HuR deletion promoted atherosclerosis by inducing defective autophagy.

## Results

### HuR levels were reduced in atherosclerotic plaque

**Fig. 1:**
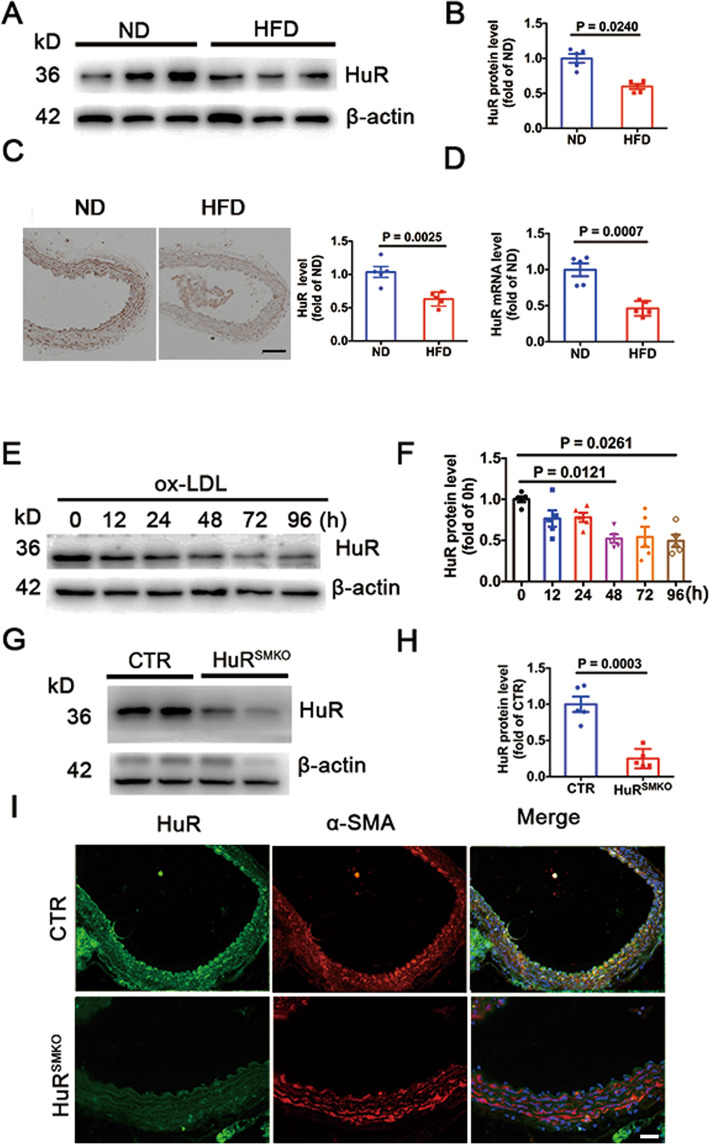
HuR levels were reduced in atherosclerotic plaque. **A**, **B** Western blot analysis of HuR protein level in aortas from apolipoprotein E-knockout (ApoE^−/−^) mice fed a high-fat diet (HFD) or normal diet (ND) (*n* = 5). **C** Immunohistochemical staining of HuR in aortas from ApoE^−/−^ mice fed an HFD or ND (*n* = 5). **D** Quantitative RT-PCR analysis of HuR mRNA level in aortas from ApoE^−/−^ mice fed an HFD or ND (*n* = 5). **E**, **F** Western blot analysis of HuR protein level in vascular smooth muscle cells (VSMCs) induced by 50 μg/ml oxidized low-density lipoprotein (ox-LDL) at different times (*n* = 5). **G**, **H** Western blot analysis of HuR protein level in aortas from control and HuR^SMKO^ mice (*n* = 5). **I** Immunofluorescent staining of aortas from control and HuR^SMKO^ mice to determine HuR (green) and α-SMA (α-smooth muscle actin; red) localization. Scale bar = 50 μm.

To investigate the role of HuR in atherosclerosis, we fed ApoE^−/−^ mice with an ND or HFD for 12 weeks. HuR protein level was decreased in aortas from the HFD group (Fig. 1A, B). As compared with the ND group, for the HFD group, HuR was downregulated in atherosclerotic lesions (Fig. 1C). As well, HuR mRNA level was significantly reduced in aortas from the HFD group (Fig. 1D). HuR protein level was decreased in VSMCs induced by ox-LDL at 24, 48, 72, and 96 h (Fig. 1E, F). For further study, we used HuR^SMKO^ mice generated from a hybrid of HuR-floxed mice and α-SMA-Cre transgenic mice. HuR protein level was downregulated in aortas from HuR^SMKO^ mice (Fig. 1G, H). HuR was not expressed in the aortic smooth muscle layer of HuR^SMKO^ mice as compared with control mice (Fig. 1I). In summary, the expression of HuR in atherosclerotic plaques was decreased, which suggests that HuR acts on atherosclerosis.

### HuR deletion in smooth muscle exacerbated atherosclerosis

**Fig. 2:**
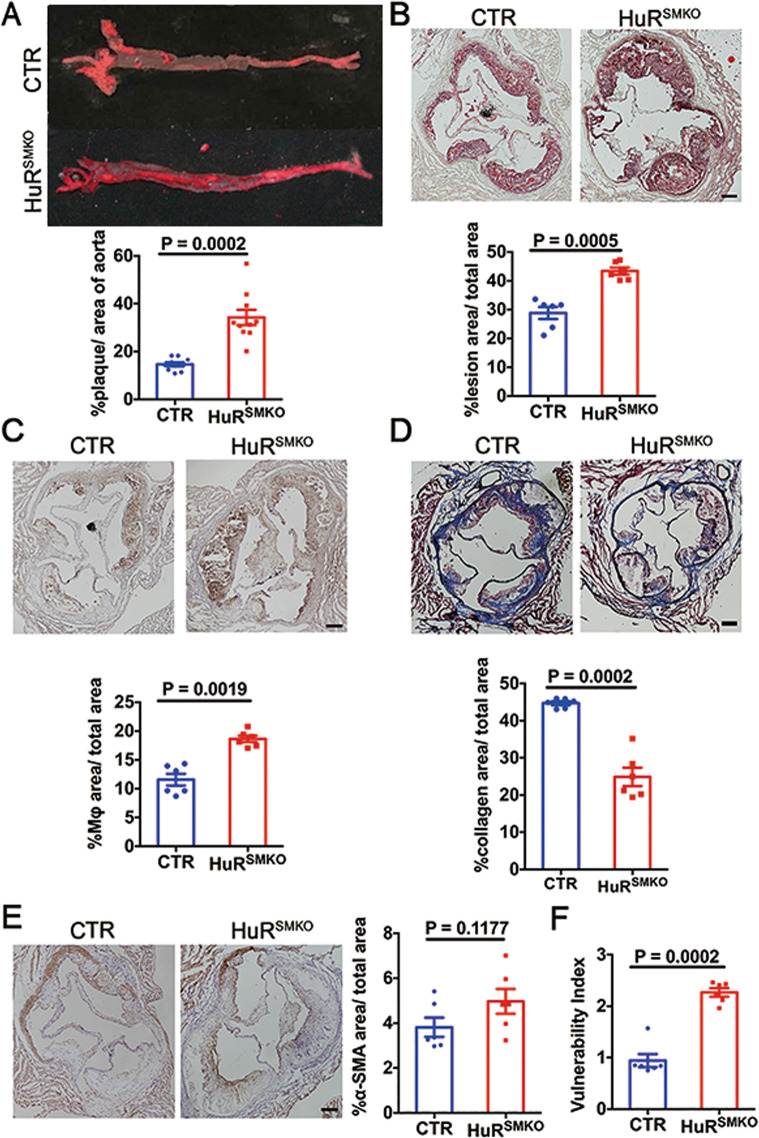
HuR deletion in smooth muscle cells exacerbated atherosclerosis. CTR and HuR^SMKO^ mice were injected with rAAV/D377Y-mPCSK9 and fed a Paigen diet for 12 weeks. **A** Oil-red O staining in aortas (*n* = 10). **B** Oil-red O staining in aortic roots (*n* = 6). Scale bar = 200 μm. **c** Immunohistochemical staining of MOMA-2 in aortic roots (*n* = 6). Scale bar = 200 μm. **D** Masson staining in aortic roots (*n* = 6). Scale bar = 200 μm. **E** Immunohistochemical staining of α-SMA (*n* = 6). Scale bar = 200 μm. **F** Vulnerable index of atherosclerotic plaques (*n* = 6).

To explore the role of smooth-muscle HuR in atherosclerosis, control, and HuR^SMKO^ mice were injected with rAAV/D377Y-mPCSK9 then fed a Paigen diet for 12 weeks. The proportion of atherosclerotic surface lesions was greater in HuR^SMKO^ than control mice (33.98 ± 6.56% vs 14.68 ± 2.47%, *p* < 0.001) (Fig. 2A). Oil-red O-stained aortic roots showed significantly increased lesion area in HuR^SMKO^ than control mice (43.38 ± 2.75% vs 28.80 ± 4.66%, *p* < 0.01) (Fig. 2B). Furthermore, deletion of HuR increased macrophage accumulation (Fig. 2C) and matrix metalloproteinase 2 (MMP2) level (Supplementary Fig. 2B), decreased collagen content (Fig. 2D). However, VSMC content and total monocyte/macrophages did not differ from controls (Fig. 2E and Supplementary Fig. 2A). From the above results, we calculated the plaque vulnerability index, which was elevated after HuR deficiency (Fig. 2F). Taken together, lack of HuR in VSMCs promoted the development of atherosclerosis.

### Loss of HuR promoted apoptosis in atherosclerosis

**Fig. 3:**
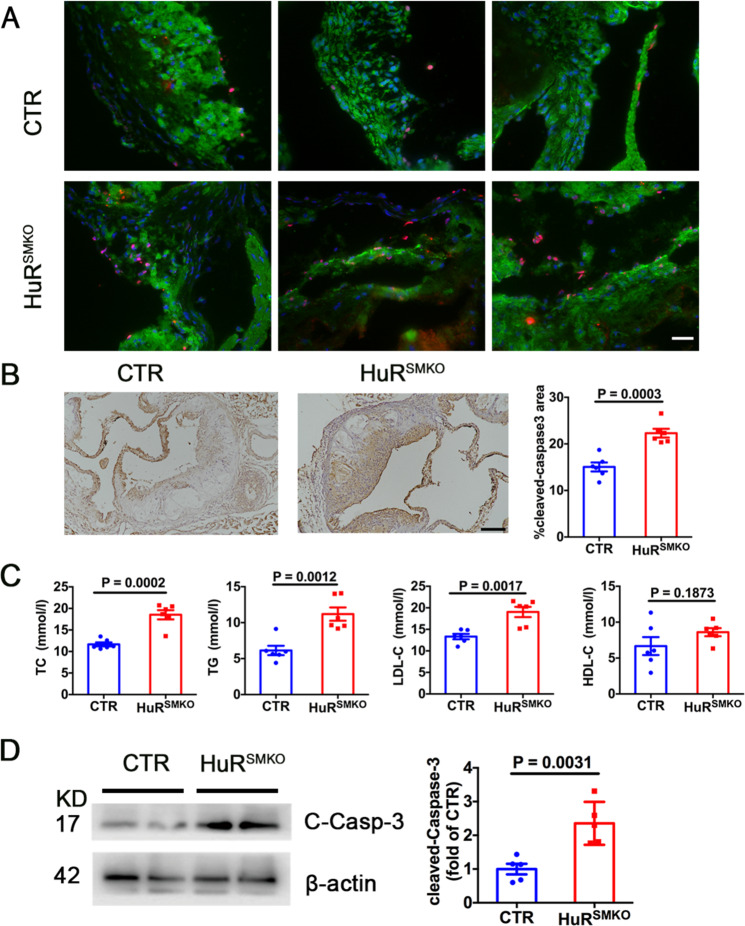
Loss of HuR promoted apoptosis in atherosclerosis. **A** Immunofluorescent staining of aortic roots from CTR and HuR^SMKO^ mice to determine TUNEL-positive VSMCs. Red puncta denotes TUNEL-positive cells. Green region denotes α-SMA. Scale bar = 20 μm. **B** Immunohistochemical staining of cleaved caspase-3 in aortic roots (*n* = 6). Scale bar = 50 μm. **C** Serum lipid profiles (total cholesterol [TC], triglycerides [TG], high-density lipoprotein cholesterol [HDL-C], and low-density lipoprotein cholesterol [LDL-C]) (*n* = 6). **D** Western blot analysis of cleaved caspase-3 in control and HuR-deficient SMCs (*n* = 5).

Numerous studies have confirmed the existence of apoptosis in atherosclerotic plaques, which even affects the stability of plaques^7,23^. To determine whether HuR knockout affected apoptosis, aortic root sections underwent TUNEL staining. Loss of HuR markedly increased the TUNEL-positive SMCs in HuR^SMKO^ versus control mice (Fig. 3A). Meanwhile, the expression of the apoptosis-related protein cleaved caspase-3 was upregulated in aortic roots of HuR^SMKO^ mice (Fig. 3B). Besides, HuR deletion also increased the level of cleaved caspase-3 in SMCs (Fig. 3D). Also, the serum levels of TC, TG, LDL-C were higher in HuR^SMKO^ than control mice (Fig. 3C). To learn why HuR^SMKO^ mice have the phenotype of dyslipidemia, HuR expression in hepatocytes and liver fibroblasts were detected. Results showed that HuR expression was no difference in hepatocytes, but decreased in liver fibroblasts from HuR^SMKO^ mice compared with control (Supplementary Fig. 1A, B). We further examined serum aspartate transaminase (AST) and alanine transaminase (ALT) levels and found elevated AST level from HuR^SMKO^ mice (Supplementary Fig. 1C, D). Thus, HuR deficiency in smooth muscle increased apoptosis in atherosclerotic mice, and loss of HuR in liver fibroblasts may contribute to hepatic dysfunction and dyslipidemia.

### HuR deletion resulted in defective autophagy

**Fig. 4:**
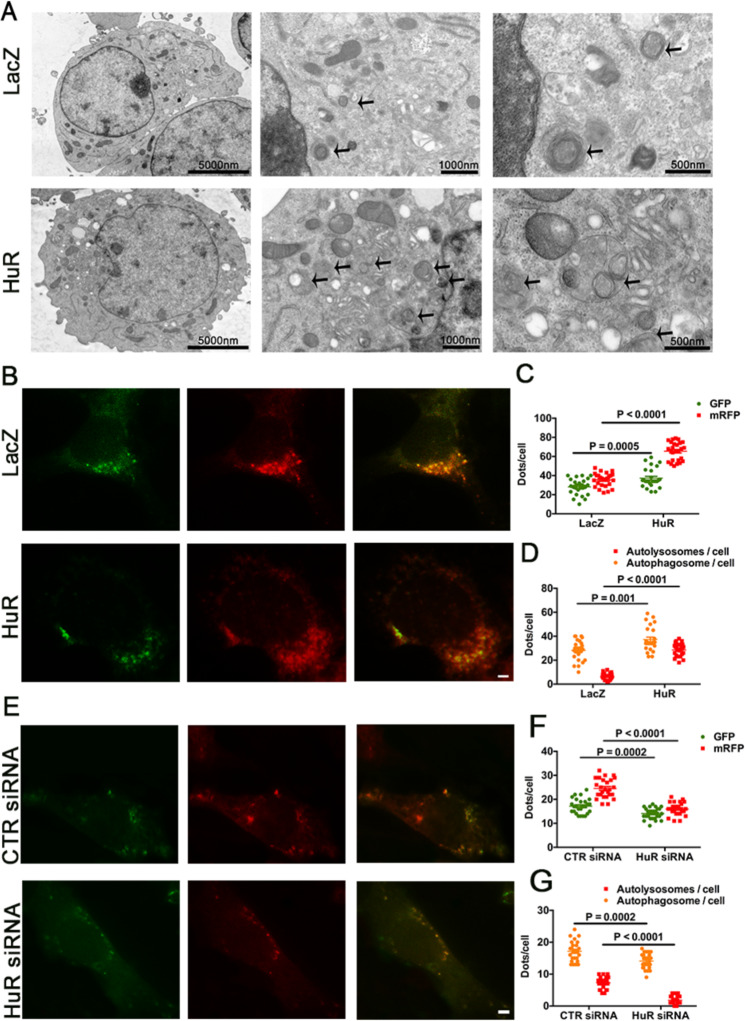
HuR deletion resulted in defective autophagy. **A** Transmission electron microscopy of autophagosomes (black arrow) in VSMCs infected with ad-HuR or ad-LacZ. Scale bar = 5000 nm (left), 1000 nm (middle), 500 nm (right). **B**–**D** fluorescence photomicrographs (**B**) and quantification (**C**, **D**) of GFP-mRFP-LC3II puncta in VSMCs infected with ad-HuR or ad-LacZ (*n* = 26). Scale bar = 10 μm. **E**–**G** Fluorescence photomicrographs (**E**) and quantification (**F**, **G**) of GFP-mRFP-LC3II puncta in VSMCs transfected with control or HuR siRNA (*n* = 26). Scale bar = 10 μm. Yellow puncta denotes autophagosome. Red puncta denotes autolysosome.

VSMC autophagy was reported to be important during the process of atherosclerosis^14,15^. To detect whether HuR could regulate autophagy, we used transmission electron microscopy after VSMCs were infected with ad-HuR or ad-LacZ for 48 h. HuR overexpression increased the number of autophagosomes (Fig. 4A). Also, autophagic flux was monitored in VSMCs by infection with ad-GFP-mRFP-LC3II. As compared with controls, VSMCs with HuR overexpression by ad-HuR infection showed an increased number of GFP^+^/RFP^+^ and GFP^−^/RFP^+^ LC3II puncta (Fig. 4B–D). However, VSMCs knocked down by HuR siRNA transfection showed decreased number of GFP^+^/RFP^+^ and GFP^−^/RFP^+^ LC3II puncta as compared with controls (Fig. 4E–G). Thus, HuR overexpression induced autophagic flux, and loss of HuR suppressed autophagic flux and resulted in defective autophagy.

### AMPKα1 and AMPKα2 were the target genes of HuR

**Fig. 5:**
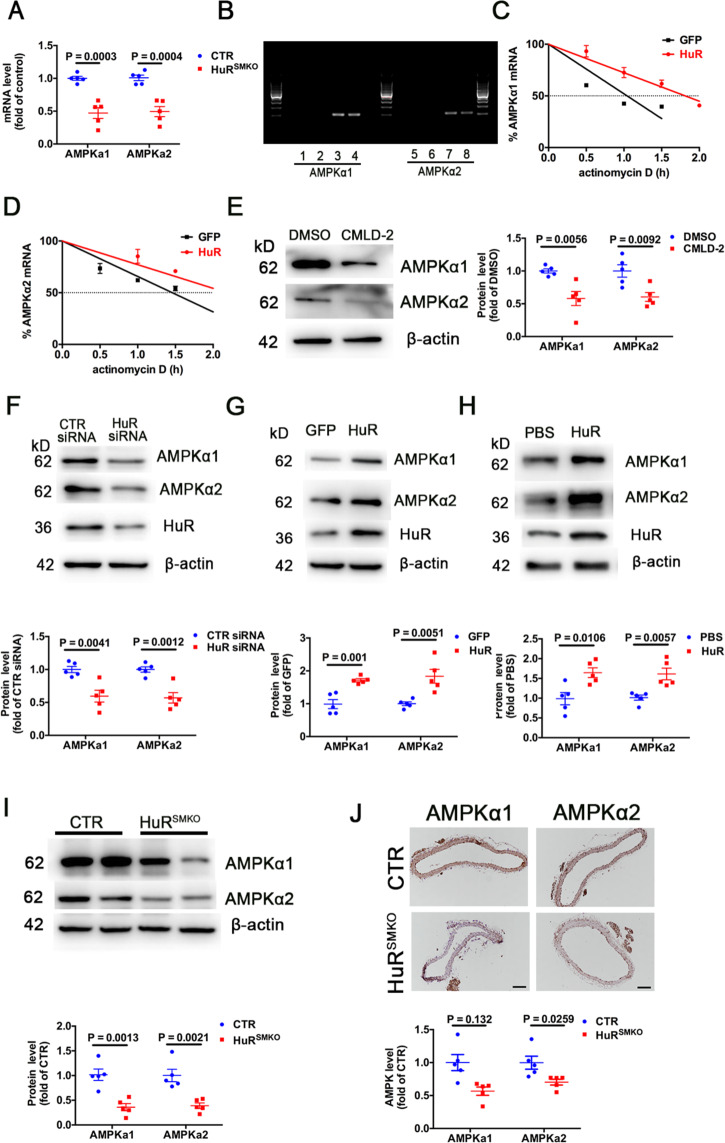
AMPKα1 and AMPKα2 are the target genes of HuR. **A** Quantitative RT-PCR analysis of aortic mRNA levels of AMPKα1, AMPKα2 from CTR and HuR^SMKO^ mice (*n* = 5). **B** RNA immunoprecipitation with anti-HuR or control IgG antibody. Lanes 1, 5, no template PCR control; lanes 2, 6, IgG RNA immunoprecipitation; lanes 3, 7, anti-HuR RNA immunoprecipitation; lanes 4, 8, 10% input. **C**, **D** VSMCs were infected with adenovirus-expressing GFP (green fluorescent protein) or HuR and then treated with actinomycin D (5 μg/ml). Quantified RT-PCR analysis of percentage mRNA levels of AMPKα1 (**C**) and AMPKα2 (**D**) (*n* = 5) in VSMCs. **E** Western blot analysis of AMPKα1 and AMPKα2 in VSMCs treated with DMSO or 30 μM CMLD-2 for 24 h (*n* = 5). **F** Western blot analysis of AMPKα1 and AMPKα2 in VSMCs transfected with CTR siRNA or HuR siRNA for 48 h (*n* = 5). Western blot analysis of AMPKα1 and AMPKα2 in VSMCs, **G** infected with ad-GFP or HuR (*n* = 5), and **H** treated with PBS or 0.5 μg/μl recombinant HuR protein for 48 h (*n* = 5). **I** Western blot analysis of AMPKα1 and AMPKα2 in aortas from CTR and HuR^SMKO^ mice (*n* = 5). **J** Immunohistochemical staining of AMPKα1 and AMPKα2 in aortas from CTR and HuR^SMKO^ mice (*n* = 5). Scale bar = 100 μm.

As a key factor in cellular energy metabolism and activator of autophagy, AMP-activated protein kinase (AMPK) participates in various physiological and pathological processes^24^. The mRNA levels of AMPKα1 and AMPKα2 were decreased in aortas from HuR^SMKO^ mice (Fig. 5A). To detect whether AMPKα was the HuR target gene, the sequence of mouse AMPKα transcripts was analyzed and there are 3 AREs in the 3′ UTR of AMPKα1 and 4 AREs in the 3′ UTR of AMPKα2. Next, we examined the binding of HuR to AMPKα1 and AMPKα2 mRNA by RNA immunoprecipitation and mRNA stability assay. HuR could bind to the mRNAs of AMPKα1 and AMPKα2 (Fig. 5B). Meanwhile, the stability of AMPKα1 and AMPKα2 mRNAs was increased by HuR overexpression (Fig. 5C, D), but decreased by HuR deficiency (Supplementary Fig. 3A, B). Then we further verified the relationship between HuR and AMPKα. VSMCs were stimulated with 30 μM CMLD-2, a HuR inhibitor, for 24 h. The protein expression of AMPKα was sharply reduced in VSMCs with CMLD-2 treatment (Fig. 5E) and AMPKα1 and AMPKα2 protein levels in VSMCs were reduced after HuR knockdown by HuR siRNA transfection (Fig. 5F). In contrast, the levels of AMPKα1 and AMPKα2 were increased in VSMCs with HuR overexpression by ad-HuR infection or HuR recombinant-protein stimulation (Fig. 5G, H). The protein levels of AMPKα1 and AMPKα2 were significantly reduced in HuR^SMKO^ versus control mice (Fig. 5I). Immunohistochemical staining results for AMPKα1 and AMPKα2 in aortas from control and HuR^SMKO^ mice were consistent with the above results (Fig. 5J). The effect of HuR on AMPK expression was further confirmed by oxLDL-stimulated SMCs after HuR knockdown or expression (Supplementary Fig. 4A, B). Therefore, AMPKα1 and AMPKα2 are the target genes of HuR.

### HuR positively regulates autophagy

**Fig. 6:**
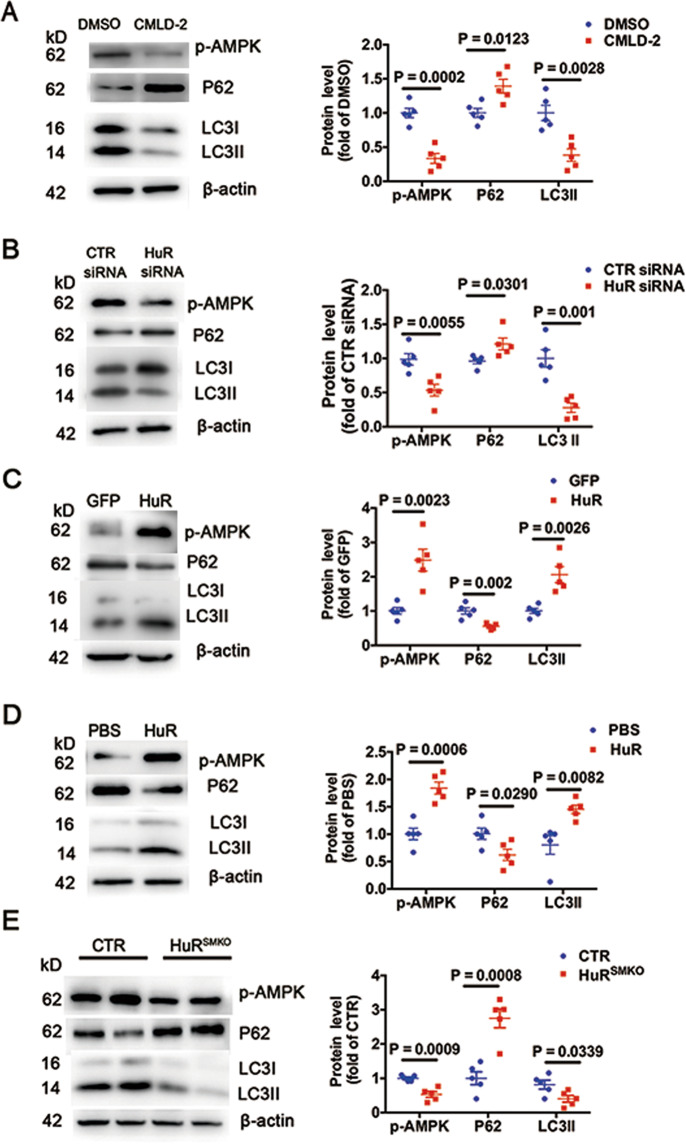
HuR positively regulates autophagy. Western blot analysis of p-AMPK, p62, and LC3II in VSMCs. **A** Treated with DMSO or 30 μM CMLD-2 for 24 h (*n* = 5). **B** Transfected with CTR or HuR siRNA for 48 h (*n* = 5). **C** Infected with ad-GFP or ad-HuR (*n* = 5), and **D** treated with PBS or 0.5 μg/μl recombinant HuR protein for 48 h (*n* = 5). **E** Western blot analysis of p-AMPK, p62, and LC3II in aortas from CTR and HuR^SMKO^ mice (*n* = 5).

Because AMPK could induce autophagy^25^ and AMPKα is the HuR target gene, we investigated whether HuR regulates autophagy. HuR inhibition with CMLD-2 or HuR siRNA decreased levels of p-AMPK and LC3II and increased p62 level in VSMCs (Fig. 6A, B). In contrast, levels of p-AMPK and LC3II were elevated and that of p62 was decreased with HuR overexpression by ad-HuR infection or HuR recombinant-protein stimulation (Fig. 6C, D). Furthermore, as compared with control mice, HuR^SMKO^ mice showed lower levels of p-AMPK and LC3II and higher level of p62 (Fig. 6E). Thus, HuR positively regulates autophagy.

### Pharmacological AMPK activation induced autophagy and suppressed atherosclerosis in HuR^SMKO^ mice

**Fig. 7:**
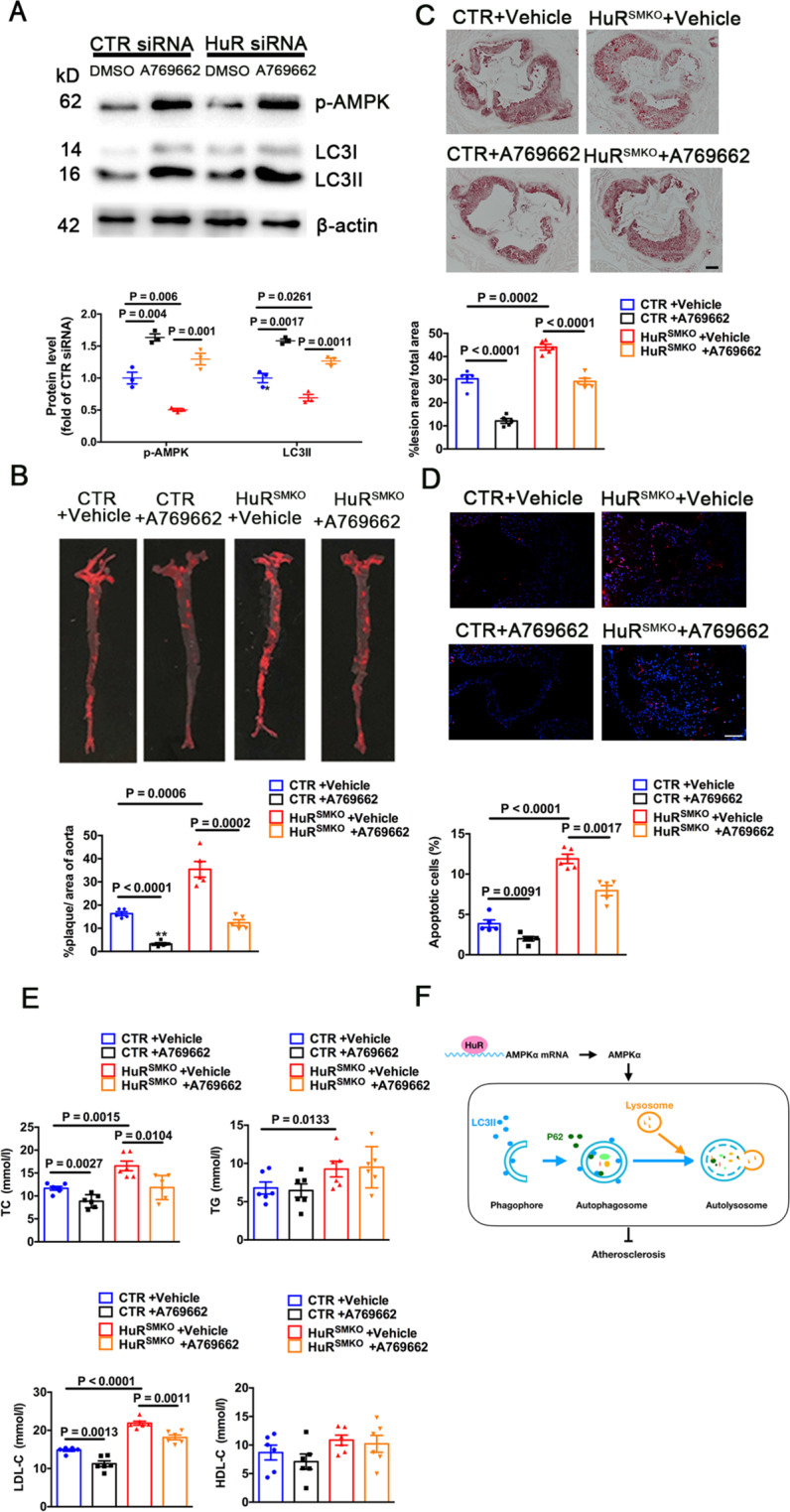
Pharmacological AMPK activation induced autophagy and suppressed atherosclerosis in HuR^SMKO^ mice. **A** Western blot analysis of VSMCs transfected with CTR or HuR siRNA for 48 h, then treated with 200 μM A769662 for 1 h (*n* = 5). CTR and HuR^SMKO^ mice were single injected with rAAV/D377Y-mPCSK9, then intraperitoneally injected with 30 mg/kg A769662 daily and fed a Paigen diet for 12 weeks. **B** Oil-red O staining in aortas (*n* = 5). **C** Oil-red O staining in aortic roots (*n* = 5). Scale bar = 200 μm. **D** TUNEL staining in aortic roots (*n* = 5). Scale bar = 50 μm. **E** TC, TG, HDL-C, and LDL-C levels (*n* = 6). **F** Diagram for the role of smooth-muscle HuR in atherosclerosis via AMPK-mediated autophagy. HuR indicates human antigen R; AMPK, Adenosine 5′-monophosphate-activated protein kinase.

To further demonstrate that HuR regulates autophagy via AMPK, VSMCs were transfected with control or HuR siRNA then treated with the AMPK activator A769662. The levels of p-AMPK and LC3II were increased with A769662 in control and HuR-deficient VSMCs (Fig. 7A). In animal experiments, control and HuR^SMKO^ mice were given an intraperitoneal injection of A769662 daily and fed a Paigen diet for 12 weeks after single intravenous injection of rAAV/D377Y-mPCSK9. The plaque area was significantly decreased after A769662 treatment in control and HuR^SMKO^ mice (Fig. 7B, C). Also, A769662 reduced the number of apoptotic cells (Fig. 7D). Furthermore, only TC and LDL-C blood levels were slightly reduced with A769662 treatment (Fig. 7E). Thus, smooth-muscle HuR protects against the development of atherosclerosis via AMPK-mediated autophagy.

## Discussion

In this study, the expression of HuR was decreased in atherosclerotic plaques from ApoE^−/−^ mice with an HFD for 12 weeks. However, Rudolf Pullmann and Mitali Ray reported that increased HuR expression in atherosclerotic plaques from patients with neointimal proliferation and LDLR^−/−^ mice lacking of IL-19^26,27^. These results suggested that HuR may play an important and complicated role in atherosclerosis. To explore the potential role of HuR in atherosclerosis, we generated smooth muscle-specific HuR knockout mice and constructed an atherosclerotic model. As compared with controls, HuR^SMKO^ mice more frequently exhibited atherosclerotic plaques and increased instability of plaques. Knockout of HuR inducing atherosclerosis was attributed to defective autophagy. Mechanically, HuR could bind to and stabilize the mRNAs of AMPKα1 and AMPKα2, thereby increasing the expression of AMPKα and enhancing autophagy (Fig. 7F).

As a member of RNA-binding proteins, HuR has a primary role to bind to the target mRNAs and modulate their stability and translational efficiency^28^. HuR also interacts with other types of RNA, including small interfering RNA, long noncoding RNAs, and circular RNAs^29^. Binding to RNAs such as cyclinA, hypoxia-inducible factor-1, cyclooxygenase 2, B-cell lymphoma-2 is associated with proliferation and apoptosis; thus, HuR is related to the occurrence of tumorigenesis^30,31^. HuR regulates mRNAs of inflammatory factors such as interleukin 6, so HuR may be involved in inflammatory diseases^28,32^. In addition, HuR is also involved in metabolic diseases such as diabetes by binding to glucose transporter (GLUT1) mRNA^33^. After the discovery of the relationship between HuR and hypertension in VSMCs^22^, here we continued to use smooth muscle-specific HuR knockout mice to explore the role of HuR in atherosclerosis. Smooth-muscle HuR protected against the development of atherosclerosis by targeting AMPKα, which expands our understanding of HuR in cardiovascular diseases.

AMPK is a serine/threonine-protein kinase that acts as a central component of the signaling pathway regulating the conversion between anabolism and catabolism^34^. AMPK is a heterotrimer composed of one catalytic subunit and two regulatory subunits. The AMPK subunits all have multiple isoforms. Differences in isoform composition affects AMPK localization and function. Catalytic subunit α, which has two different isoforms, α1 and α2, is a major functional component of AMPK activation. Phosphorylation at Thr172 of α subunit is essential for AMPK activation^35^. Because mammalian AMPK is sensitive to the AMP:ATP ratio, any cellular process that reduces ATP levels or increases AMP concentration could activate AMPK. Some cytokines such as leptin, adiponectin, and ghrelin and some drugs such as AICAR, A769662, and metformin can activate AMPK directly or indirectly^36^. Also, AMPK activation is known to be mediated by liver kinase B1 (LKB1), Ca^2+^/calmodulin-dependent kinase CaMKK2 (CaMKKβ), and transforming growth factor-β-activated kinase 1 (TAK1)^24,36,37^. Some studies have reported that ΔNp63α, a p53 family member, and microRNAs such as mir-19 and mir-101 can regulate the expression of AMPKα^38–40^. Our study found that HuR could regulate AMPKα, which enhances our understanding of the regulatory mechanisms of AMPKα expression and activation. Recent studies showed that AMPK plays an important role in lipid metabolism^41,42^. In this study, we demonstrated that AMPKα1 and AMPKα2 were the target genes of HuR. Therefore, we speculated that HuR may regulate lipid metabolism through AMPK in hepatocytes. In the future study, hepatocyte-specific HuR knockout mice will be generated and these mice are predicted to have the phenotype with dyslipidemia. Whether and how HuR regulating the impact of AMPK on dyslipidemia will be explored.

Autophagy is a major intracellular degradation system that aims to dynamically cycle energy and matter for cell renewal and homeostasis^43^. The most important upstream regulators of autophagy are AMPK and mammalian target of rapamycin. Moreover, some studies have found that AMPK and mammalian target of rapamycin interact with each other^44,45^. From autophagosomes to autolysosomes, autophagy-related proteins such as Atg5, Atg7, LC3II, and p62 are indispensable for the occurrence and development of autophagy^46^. In recent years, the relationship between autophagy and atherosclerosis has also attracted much attention. Macrophage-specific Atg5 deficiency promotes atherosclerosis by interfering with cholesterol transport, apoptosis, and inflammation^47–49^. Endothelial-specific Atg5 and Atg7 deletion cause atherosclerotic plaque formation^50,51^. Knockout of Atg7 in VSMCs accelerates the process of atherosclerosis and promotes the regeneration of intima after injury^15^. Autophagy is closely related to cell senescence and apoptosis, which are also involved in atherosclerosis. In this study, knockout of HuR led to defective autophagy, which further increased apoptosis and atherosclerosis.

In summary, HuR could increase AMPK-mediated smooth muscle autophagy and play a protective role in atherosclerosis. Our research provides a new concept and drug target for treating atherosclerosis.

## Materials and methods

### Reagents

Adenovirus expressing GFP, HuR (ad-HuR, ad-GFP), and recombinant adeno-associated viral of murine proprotein convertase subtilisin/kexin type 9 mutants (rAAV/D377Y-mPCSK9) were from Vigenebio (MD, USA). Adenovirus expressing LacZ (ad-LacZ) and GFP-mRFP-LC3II (ad-GFP-mRFP-LC3II) were from Hanbio (Shanghai). Actinomycin D and Oil-red O were from Sigma (St Louis, MO, USA). A769662 was from Selleck Chemical (Houston, TX, USA). Ox-LDL was from Yiyuan (Guangzhou, China). CMLD-2 was from Millipore (Temecula, CA, USA). Recombinant HuR protein was from Proteintech (Chicago, IL, USA). Control and HuR siRNA were synthesized by BioSune (Shanghai). The sequences for HuR siRNA were 5′-CCAGUUUCAAUGGUCAUAATT-3′ and 5′-UUAUGACCAUUGAAACUGGTT-3′ and control siRNA were 5′-GGUUGAAUCUGCAAAGCUUTT-3′ and 5′-AAGCUUUGCAGAUUCAACCTT-3′. Paigen diet was from Trophic Diets (TP28640, China), containing 15% fat, 0.5% bile salt, and 1.25% cholesterol.

### Cell culture and infection

Mouse smooth muscle cells were from ATCC (Manassas, VA, USA) with STR authentication. Cells were cultured in DMEM containing 10% fetal bovine serum and 50 μg/ml penicillin/streptomycin and seeded in 6-well plates at 1.0 × 10^4^ cells/cm^2^. VSMCs grown to 70% confluence were infected with adenovirus at multiplicity of infection 75 for 48 h.

### Mouse models

Smooth muscle-specific HuR knockout (HuR^SMKO^) mice were generated as described^22^. Male control and HuR^SMKO^ mice at 8 weeks old were given a single tail-vein injection with rAAV/D377Y-mPCSK9 at 1.5 × 10^11^ pfu for each mouse as described^52^ and fed a Paigen diet for 12 weeks. Male apolipoprotein E-deficient mice (ApoE^−/−^) at 8 weeks old were from Vital River (Beijing) and were divided into two groups for high-fat diet (HFD) or normal chow diet (ND) feeding. Mice were housed at 25 °C, 12-h light/dark. Mice were euthanized using profound anesthesia with 4% isoflurane followed by exsanguination and tissue removal. The animal experiment was approved by the Animal Care Committee of Shandong University and was performed in compliance with the Animal Management Rules of the Chinese Ministry of Health. All animal experiments were performed conform the guidelines from Directive 2010/63/EU of the European Parliament on the protection of animals used for scientific purposes.

### Atherosclerotic lesion assay

Mice were euthanized and perfused with PBS. Hearts and aortas from the proximal ascending aorta to the abdominal aorta were removed and fixed in 4% paraformaldehyde. Aortas were dissected free of fat and adventitial tissue, opened longitudinally, stained with 0.5% freshly-made Oil-red O for 2 h, and pinned onto black silicon plates for imaging. After optical cutting temperature (OCT) compound embedding, the heart was cut to the aortic root to reveal the valvular lobe, stained with 0.5% Oil-red O and observed by microscopy. The results were reported as percentage of lesion area to total aortic area.

### Morphology of aorta and aortic root

Aortas and hearts were embedded in OCT and sliced into 5-μm-thick frozen sections. The heart was cut to the aortic root. After sections were hydrated, immunofluorescence, immunohistochemistry, or other special staining was performed. To detect HuR-knockout efficiency in VSMCs, fluorescent double labeling of aortic sections was performed with HuR antibody (1:300, Millipore) and α-SMA antibody (1:300, Abcam). Sections of aortic roots were immunostained with MOMA-2 antibody (1:300, Abcam) to detect macrophage content and α-SMA antibody to detect VSMC content. The collagen of aortic root was detected by using a Masson staining kit (Solarbio, Beijing). The plaque vulnerability index was calculated as follows: (macrophage staining % + lipid staining %)/(SMC staining % + collagen staining %)^53^.

### Detection of apoptosis

Apoptosis was detected by immunohistochemical staining with cleaved caspase-3 antibody (1:300, Affinity) and terminal UTP nick end-labeling (TUNEL). Briefly, aortic root sections were fixed in fresh 4% paraformaldehyde for 20 min at 15–25 °C, then incubated with premeabilization solution containing 0.1% Triton X-100 and 0.1% sodium citrates for 2 min at 2–8 °C. Staining followed the recommendations of the in-situ Cell Death Detection Kit (Roche, Basel, Switzerland).

### Lipid profile assays

Serum was isolated for determining levels of total cholesterol (TC), triglycerides (TG), high-density lipoprotein cholesterol (HDL-C), and low-density lipoprotein cholesterol (LDL-C). The blood lipid assay kit was from Jiancheng Bioengineering Institute (Nanjing, China).

### Western blot analysis

A 15-μg amount of protein lysates from isolated aorta and SMCs were run on a 12% SDS-PAGE gel and immunoblotted overnight with the primary antibodies for HuR (29 ng/ml, 12582S, CST), AMPKα1 (1 μg/ml, Ab32047, Abcam), AMPKα2 (1 μg/ml, ab3760, Abcam), p-AMPK (27 ng/ml, 2535S, CST), p62 (293 ng/ml, 18420-1-AP, Proteintech), LC3I/II (64.5 ng/ml, 4108S, CST), Cleaved Caspase-3 (1 μg/ml, BF0711, Affinity), and β-actin (1 μg/ml, 20536-1-AP, Proteintech).

### RNA immunoprecipitation assay

Whole-cell lysates were incubated overnight at 4 °C with protein A/G beads pre-conjugated with 5 μg rabbit IgG or HuR antibody (Millipore). RNA was then isolated from immunoprecipitates by using the Magna RIP kit (Millipore) with the primer sequences AMPKα1: 5′-GGCACCTTCGGGAAAGTGAA-3′ and 5′-TGTGAGGGTGCCTGAACAGC-3′ and AMPKα2: 5′-GAAGATTCGCAGTTTAGATGTTG-3′ and 5′-TCGAACAATTCACCTCCAGA-3′.

### GFP-mRFP-LC3II punctation

VSMCs were infected with ad-GFP-mRFP-LC3II for 48 h. LC3II spots were observed by fluorescence microscopy (Nikon, Tokyo). GFP-RFP-LC3II serves as a specific marker for autophagic flux that relies on the different nature of GFP and RFP fluorescence under acidic conditions. Because GFP was quenched in the lysosomal acidic conditions, autophagosomes are shown as yellow puncta (RFP^+^GFP^+^), and autolysosomes are shown as red puncta (RFP^+^GFP^−^) in green- and red-merged images^54^. The mean number of LC3II points per cell was calculated from more than 30 cells.

### Transmission electron microscopy

After infection with ad-LacZ or HuR for 48 h, VSMCs were harvested and fixed in electron microscope fixative containing 2.5% glutaraldehyde (Servicebio, Wuhan, China) for 2 h. Samples were fixed in 1% osmium tetroxide and dehydrated with ethanol in ascending order, followed by epoxide resin-embedding and cut into sections. Images of stained sections were collected by transmission electron microscopy (HT7700, Hitachi, Japan).

### Statistical analysis

Group allocation for the experiments was randomized and not blinded. Sample analyses were not blinded. Data are expressed as mean ± SEM and were analyzed by using GraphPad Prism 6.0. All data were tested for normal distribution and equal variances. Student *t* test was used to compare two groups with 95% confidence interval. One-way ANOVA and Bonferroni post-tests were used in four groups. Statistical significance was set at *P* < 0.05.

## Supplementary information

Supplemental Material
