# Supplementary material for: Smooth muscle-specific HuR knockout induces defective autophagy and atherosclerosis
Source: Cell Death Dis. 2021 Apr 9;12(4):385. doi: 10.1038/s41419-021-03671-2 (PMC8035143; doi:10.1038/s41419-021-03671-2)
Supplement: Supplementary file 1 — Supplemental Material [file 41419_2021_3671_MOESM1_ESM.pdf]

Supplementary data

Smooth Muscle-specific HuR Knockout Induces Defective Autophagy and Atherosclerosis

Shanshan Liu[1,2], Xiuxin Jiang[3], Xiuru Cui[1], Jingjing Wang[4], Shangming Liu[5], Hongxuan Li[1], Jianmin Yang[1], Cheng Zhang[1], Wencheng Zhang[1,2*]

[1]The Key Laboratory of Cardiovascular Remodeling and Function Research, Chinese Ministry of Education, Chinese National Health Commission and Chinese Academy of Medical Sciences, The State and Shandong Province Joint Key Laboratory of Translational Cardiovascular Medicine, Department of Cardiology, Qilu Hospital, Cheeloo College of Medicine, Shandong University, Jinan, China; [2]Cardiovascular Disease Research Center of Shandong First Medical University, Central Hospital Affiliated to Shandong First Medical University; [3]Department of General Surgery, Qilu Hospital of Shandong University, Jinan, China; [4]Department of Physiology & Pathophysiology, School of Basic Medical Sciences, Shandong University; [5]Department of Histology and Embryology, School of Basic Medical Sciences, Shandong University.

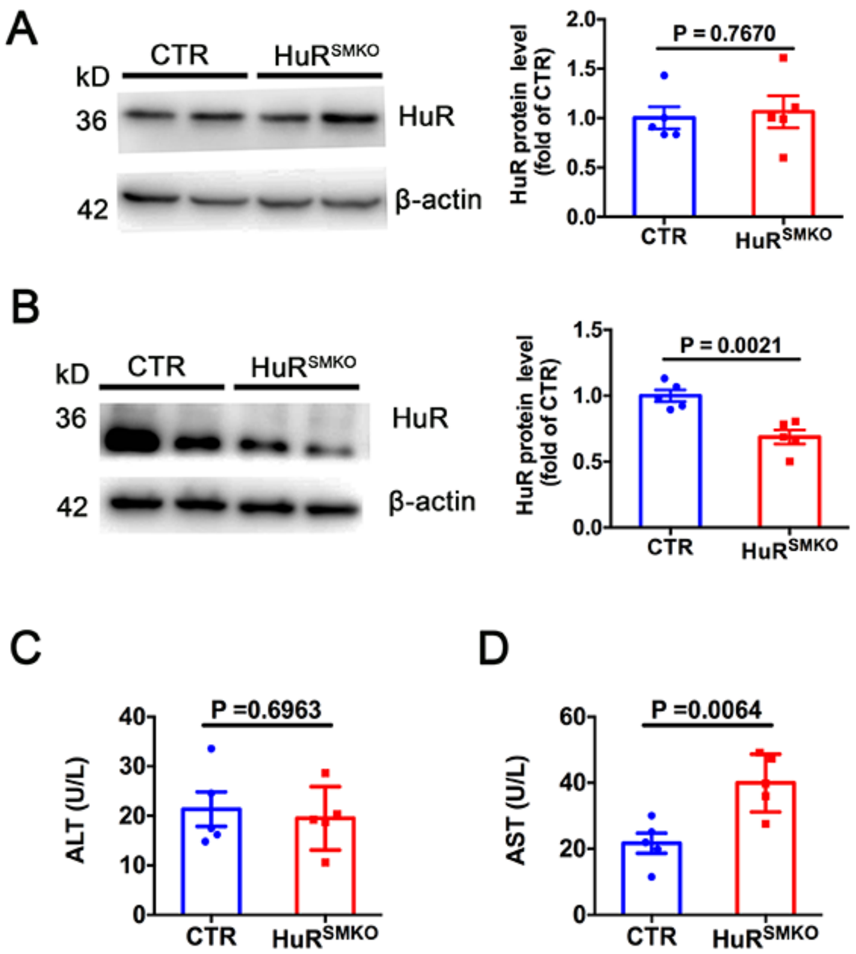

Supplementary Fig. 1. HuR expression in hepatocytes and liver fibroblasts from CTR and HuR^{SMKO} mice. A, Western blot analysis of HuR expression in hepatocytes from CTR and HuR^{SMKO} mice (n=5). **B,** Western blot analysis of HuR expression in liver fibroblasts from CTR and HuR^{SMKO} mice (n=5). **C-D,** Measurement of serum aspartate transaminase (AST) and alanine transaminase (ALT) from CTR and HuR^{SMKO} mice (n=5).

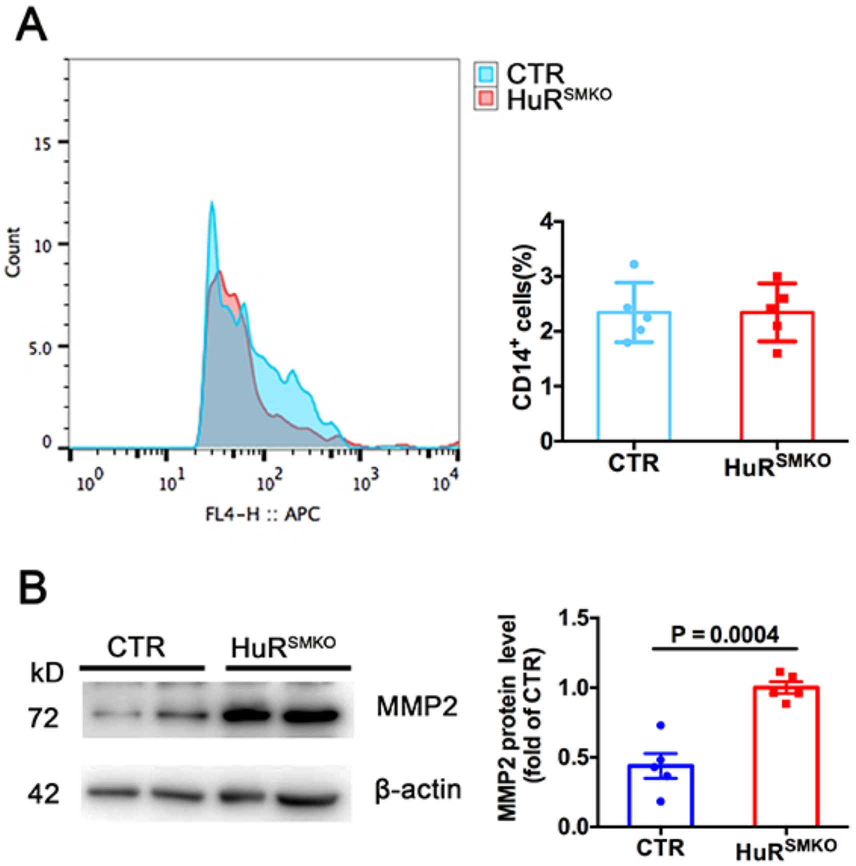

Supplementary Fig. 2. HuR deletion did not change total monocytes but increased MMP2 expression. CTR and HuR^{SMKO} mice were injected with rAAV/D377Y-mPCSK9 and fed a paigen diet. **A,** Monocytes were labeled with CD14 and then detected by whole blood flow cytometry. Representative images were shown and monocytes were analyzed (n=5). **B,** Western blot analysis of MMP2 expression in aortas from CTR and HuR^{SMKO} mice (n=5).

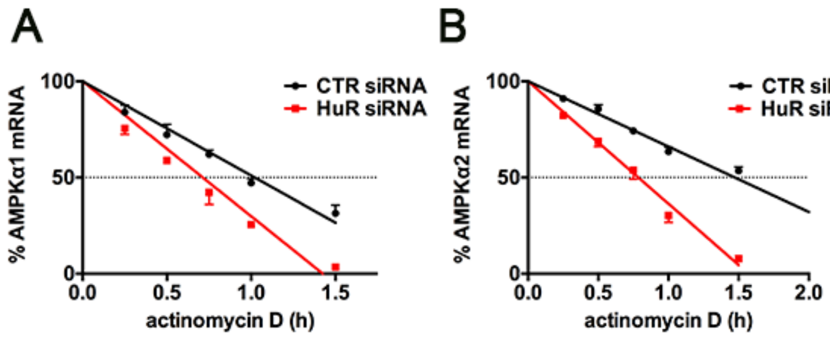

Supplementary Fig. 3. The stability of AMPKα1 and AMPKα2 mRNAs decreased by HuR deficiency. A-B, VSMCs were transfected with CTR siRNA or HuR siRNA and then treated with actinomycin D (5 μg/ml). Quantified RT-PCR analysis of mRNA levels of AMPKα1 (**A**) and AMPKα2 (**B**) in VSMCs (n=5).

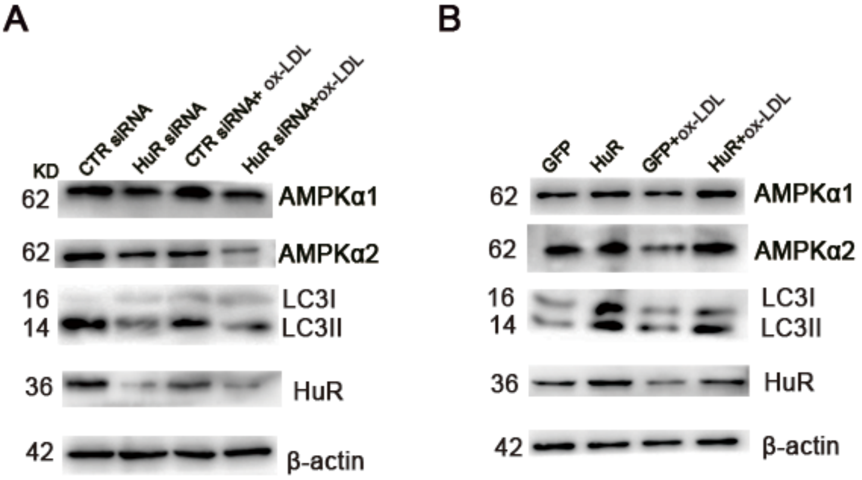

Supplementary Fig. 4. HuR regulates AMPKα expression in oxLDL-stimulated SMCs. A, Western blot analysis of AMPKα1, AMPKα2, LC3II in VSMCs transfected with CTR siRNA or HuR siRNA followed by oxLDL treatment for 24 hrs. **B,** Western blot analysis of AMPKα1, AMPKα2, LC3II in VSMCs infected with adenovirus-expressing GFP or HuR followed by oxLDL treatment for 24 hrs.
